# Evaluation of the effects of an offer of a monetary incentive on the rate of questionnaire return during follow-up of a clinical trial: a randomised study within a trial

**DOI:** 10.1186/s12874-016-0180-9

**Published:** 2016-07-15

**Authors:** Pollyanna Hardy, Jennifer L. Bell, Peter Brocklehurst, on behalf of The Epidural and Position Trial Collaborative Group, Debra Bick, Annette Briley, Edmund Juszczak, Lynn Lynch, Christine MacArthur, Phillip Moore, Mary Nolan, Felicity Plaat, Julia Sanders, Andrew Shennan, Matt Wilson, Steve Hibbert, Elizabeth Howden, Sawretse Leslie, Amber Gibney, Claire Nelis

**Affiliations:** Nuffield Department of Population Health, National Perinatal Epidemiology Unit CTU, University of Oxford, Old Road Campus, Headington, Oxford, OX3 7LF UK; Institute for Women’s Health, University College London, London, UK

**Keywords:** SWAT, Follow-up, Return rates, BUMPES, Incentive, Clinical trial, Randomised, Questionnaires

## Abstract

**Background:**

A systematic review on the use of incentives to promote questionnaire return in clinical trials suggest they are effective, but not all studies have sufficient funds to use them. Promising an incentive once data are returned can reduce the cost-burden of this approach, with possible further cost-savings if the offer were restricted to reminder letters only. This study aimed to evaluate the effect of promising a monetary incentive at first mailout versus a promise on reminder letters only.

**Methods:**

This was a randomised Study Within A Trial (SWAT) nested within BUMPES, a multicentre randomised controlled trial of maternal position in the late stage of labour in women with an epidural. The follow-up questionnaire asked for information on the women’s health, wellbeing and health service use one year following the birth of their baby. Women who consented to be contacted were randomised to a promise of a monetary incentive at first mailout or a promise on reminder letters only. Women were given an option of completing the questionnaire on paper or on online. The incentive was posted out on receipt of a completed questionnaire. The primary outcome was the overall return rate, and secondary outcomes were the return rate without any chasing from the study office, and the total cost of the vouchers.

**Results:**

A total of 1,029 women were randomised, 508 to the first mailout group and 518 to the reminder group. There was no evidence to suggest a difference between groups in the overall return rate (adjusted RR 1.03 (95 % CI 0.96 to 1.11), however the proportion returned without chasing was higher in the first mailout group (adjusted RR 1.22, 95 % CI 1.07 to 1.39). The total cost of the vouchers per participant was higher in the first mailout group (mean difference £4.56, 95 % CI £4.02 to £5.11).

**Conclusions:**

Offering a monetary incentive when a reminder is required could be cost-effective depending on the sample size of the study and the resources available to administer the reminder letters.

**Trial registration:**

The BUMPES Trial is registered with Current Controlled Trials: ISRCTN35706297, 26^th^ August 2009.

## Background

Maximising follow-up rates for postal questionnaires in randomised controlled trials is an important aspect of a well-designed and well conducted study. Loss to follow-up can lead to bias and compromise the internal and external validity of the results.

Use of monetary incentives to promote questionnaire return in clinical trials has been researched. Existing systematic reviews suggest they are effective [1, 2], but not all studies have sufficient funds to use them. Promising an incentive once data are returned can reduce the cost burden of this approach. In a systematic review Brueton et al. [2] showed evidence that an offer of a monetary incentive was comparable to the addition of a monetary incentive with the questionnaire in 2 studies with a total of 297 participants (pooled risk ratio 1.04, 95 % confidence interval 0.91 to 1.19). However, it may be possible to provide further cost-savings if the offer was restricted to the reminder letters only.

This randomised study within a trial (SWAT) was nested within the BUMPES trial, a multicentre randomised controlled trial investigating the effect of maternal position during the late stages of labour in women with an epidural. The SWAT was carried out on a population of women in the UK one year after the birth of their first child and was developed because the return rate of the follow-up questionnaire for BUMPES was lower than expected in the early stages of the trial. Since current evidence on the use of incentives includes a variety of populations, providing an evidence base on the use of incentives for postnatal women will enhance future research methodology in this population.

This SWAT aimed to evaluate the effect on the return rate of a 1-year follow-up postal questionnaire, comparing the promise of a monetary incentive at first mailout with a promise on reminder letters only.

## Methods

### Setting

This parallel group, randomised controlled SWAT nested within BUMPES was conducted on women randomised into the BUMPES study who had not already been sent their 1-year follow-up questionnaire. The questionnaire asked for information on the women’s health, wellbeing and health service use one year following the birth of their baby. Women had the option of completing the paper questionnaire and returning it using a freepost envelope, or completing it on-line using a secure web-interface.

### Participant eligibility

Women were included if they were recruited to BUMPES, consented at recruitment to receive the 1-year follow-up questionnaire, and the questionnaire had not already been sent. Women were excluded if they had had a stillbirth, their infant had died by the time of follow-up, their address details were unknown or if they were not living at the same address as their infant.

### Interventions

Women were randomly allocated to one of the following two groups: (1) an incentive cover letter sent with the first mailout of the questionnaire containing details of a promise of a £10 gift voucher (redeemable at high street shops) on return of a completed questionnaire. The covering letter included a sentence explaining that the voucher was to thank participants for their time and effort. All reminder letters included a sentence about the incentive; (2) an incentive reminder letter. For this group the cover letter sent at first mailout did not mention the incentive. If the questionnaire was not returned, all reminder letters detailed the promise of a £10 gift voucher on return of a completed questionnaire.

For both groups women were additionally contacted electronically and via text message if the contact details were available. The content of the emails and texts sent reflected the group to which the woman was randomised.

### Randomisation

Allocation was by computer random number generation and stratified by BUMPES allocation and by centre. The randomisation schedule was generated by the National Perinatal Epidemiology Unit Clinical Trials Unit and sent to the BUMPES trial office at the Comprehensive Clinical Trials Unit at University College London via a secure web-link. Randomisation to incentive cover letter at first mailout or incentive reminder letter occurred at each woman’s next follow-up point during the conduct of the BUMPES study. Each BUMPES participant was randomised once only. BUMPES trial staff were aware of the allocation due to the nature of the interventions, and the practicalities involved in sending the letters and the vouchers.

### Outcome measures

The pre-specified primary outcome measure was questionnaire return, defined as receipt of a completed or partially completed questionnaire at the BUMPES office. Pre-specified secondary outcome measures were the number of questionnaires returned without chasing by the study team and the total cost of the vouchers sent out by study arm.

### Data collection

Recording of questionnaire receipt, date received and voucher sent was made using internal trial administration systems. Postal versus online receipt was also recorded.

### Sample size

The sample size was predetermined by the numbers of questionnaires that remained to be sent at the estimated start time of the SWAT. BUMPES started recruiting in October 2010 and finished in January 2014. A total of 3,236 women were randomised. It was estimated that approximately 1,150 women would remain to be followed up at the start date of this study (beginning August 2014). Assuming that approximately 15 % of these women would be excluded from receiving the questionnaire due to stillbirth, infant death, or address details unknown or different to the infant, 980 women would be eligible to be randomised in the nested study (approximately 490 per group).

In order to assess the detectable effect size possible with the given sample size, we estimated the control group risk based on current literature. Khadjesari et al. [3] investigated the use of an offer of an incentive (a £10 Amazon gift voucher) versus no offer of an incentive on follow-up rates in an online trial. They found an increase of 9 % (95 % CI 5 % to 12 %) when using the offer of an incentive. Kenyon et al. [4] investigated the use of a monetary incentive included in reminder letters versus no incentive and found an improvement in the response rate between the two groups of 11.7 % (95 % confidence interval 4.7 to 18.6 %).

The follow-up questionnaire return rate for BUMPES up to June 2014 was 59 %. Assuming that this could increase by at least 5 % with the use of an offer of an incentive either with an incentive cover letter at first mailout or an incentive reminder letter only, a sample size of 980 would be sufficient to demonstrate an increase in questionnaire return of 8 % from 64 % in the reminder group to 72 % in the first mailout group at a two-sided 5 % significance level with 80 % power. A detectable difference of between 8 and 8.5 % would be possible for return rate estimates in the reminder group of between 60 and 70 %.

### Statistical analysis

For all analyses, an intention to treat approach was taken and participants were analysed in the groups into which they were randomly allocated, i.e. comparing outcomes for women allocated to the first mailout group with outcomes for women allocated to the reminder group, regardless of allocation received.

All analyses were based on all women randomised for whom we had data available.

Participants in the two randomised groups are described separately with respect to baseline demographics and clinical characteristics, including the primary outcome for the main BUMPES study, and recorded on the BUMPES Woman and Infant Data Collection Booklet.

The return rate and chase rate before the introduction of the randomised interventions (i.e. before the SWAT started) and at the end of the study (with both SWAT trial arms combined) is presented using numbers and percentages. The return rate and chase rate by method of completion (online versus postal) is described by trial arm using numbers and percentages.

An adjusted analysis was performed on the two return rate outcomes adjusting for centre (the stratification factor at randomisation) [5] as a random effect. The analysis was carried out using log binomial regression models, and results presented as adjusted risk ratios with 95 % confidence intervals (CI). Differences in means of the total cost averaged over the total number of participants is presented with 95 % confidence intervals.

To examine whether the effect of when vouchers are sent is consistent across specific subgroups of women, a subgroup analysis by IMD (Index of Multiple Deprivation) quintile was pre-specified. Results are presented as risk ratios plus 95 % CI for each subgroup, by intervention group, with the p value for the statistical test of interaction.

Stata/SE for Windows (version 13.1) was used for all analyses.

## Results

**Fig. 1:**
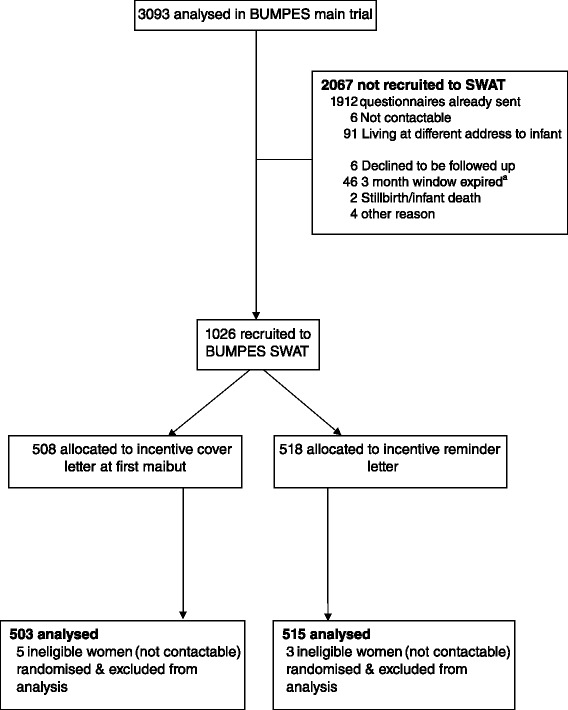
Participant flow diagram

Randomisation to the incentive nested study started on 31st July 2014 and continued until all questionnaires and reminders had been sent (last letter sent 6th March 2015). The total number of women in the SWAT was 1026. Eight women were excluded from all analyses as it was discovered after they had been randomised to the SWAT that they had moved address (see Fig. 1).

Balance between the SWAT trial arms in baseline characteristics was good. There were only small imbalances in onset of labour (spontaneous or induced), diagnosis of pre-eclampsia, and spontaneous vaginal birth (the BUMPES primary outcome) (see Table 1).

**Table 1:** Characteristics prior to study entry

Characteristic	First mailout (*n* = 503)		Reminder (*n* = 515)	
	*n*	(%)	*n*	(%)
Maternal age (years) - mean {SD} Under 20 20–24 25–29 30–34 35–39 40+	28.9 24 93 133 177 66 10	{5.6} (4.8) (18.5) (26.4) (35.2) (13.1) (2.0)	29.3 24 79 148 180 71 13	{5.5} (4.7) (15.3) (28.7) (35.0) (13.8) (2.5)
Gestational age at entry (weeks) - mean {SD} 37^+0^ – 39^+6^ 40^+0^ – 41^+6^ 42^+0^ or above	40.4 150 320 32	{1.2} (29.9) (63.8) (6.4)	40.3 167 315 32	{1.2} (32.5) (61.3) (6.2)
Index of Multiple Deprivation – quintile				
1^st^ (Least deprived) 2^nd^ 3^rd^ 4^th^ 5^th^ (Most deprived) Wales – not derived Postcode missing	64 72 83 112 95 66 11	(15.0) (16.9) (19.5) (26.3) (22.3)	72 63 91 129 88 59 13	(16.3) (14.2) (20.5) (29.1) (19.9)
Ethnic group:				
White Indian Pakistani Bangladeshi Black African Black Caribbean Any other ethnic group Not known/missing	415 20 9 2 14 7 29 7	(83.7) (4.0) (1.8) (0.4) (2.8) (1.4) (5.9)	434 14 7 1 10 2 42 5	(85.1) (2.8) (1.4) (0.2) (2.0) (0.4) (8.2)
BMI (at booking visit)				
- mean {SD} Height and/or weight not known	25.2 18	{5.2}	25.2 11	{5.3}
Onset of labour:				
Spontaneous Induced	309 193	(61.6) (38.5)	293 222	(56.9) (43.1)
Diagnosis of pre-eclampsia	12	(2.4)	22	(4.3)
Diagnosis of delay requiring intervention	266	(53.1)	272	(52.8)
Systemic opioids given prior to epidural Pethidine Diamorphine Remifentanil Morphine Meptid	142 103 38 0 0 3	(28.3) (72.5) (26.8) (0.0) (0.0) (2.1)	137 97 38 1 0 3	(26.6) (70.8) (27.7) (0.7) (0.0) (2.2)
Epidural technique:				
Epidural Combined spinal epidural	485 17	(96.6) (3.4)	496 18	(96.5) (3.5)
Woman’s pain score for last contraction - median [IQR] Missing	10 59	[0 to 32]	10 55	[0 to 30]
Able to perform straight leg raise Missing	381 29	(80.4)	408 22	(82.8)
Spontaneous vaginal birth	197	(39.2)	181	(35.2)

*SD* standard deviation, *IQR* interquartile range

Missing data are <1 % unless otherwise presented

Values are numbers (percentages) unless stated otherwise

The overall percentage of questionnaires returned before the SWAT started was considerably lower compared to that for participants included in the SWAT (1149/2067, 55.6 % vs. 743/1018, 73.0 %). This trend is also seen in the percentage returned without any reminder letters being sent (729/2067, 35.3 % vs. 476/1018, 46.8 %).

For the primary outcome, the percentage of questionnaires returned overall for those in the first mailout group was slightly higher than those in the reminder group (74.2 vs. 71.8 %), but this was not statistically significant at the 5 % level (adjusted risk ratio (RR) 1.03 and 95 % CI 0.96 to 1.11). Women who receive a cover letter promising an incentive at first mailout are more likely to return their questionnaire without a reminder letter being required compared to those receiving a standard cover letter (adjusted RR 1.22, 95 % CI 1.07 to 1.39). One in five of all questionnaires returned (152/743, 20.5 %) were completed online, with slightly fewer being returned online in the first mailout group compared to the reminder group (18.0 vs. 23.0 %). The mean difference in the total cost of the vouchers per participant was £4.56 (95 % CI £4.02 to £5.11), with the cost being higher in the group receiving the incentive cover letter at first mailout (see Table 2).

**Table 2:** Outcomes

Outcome	First mailout (*n* = 503)		Reminder (*n* = 515)		Effect measure (95 % CI)
	*n*	%	*n*	%	
Primary outcome					
Questionnaire returned Postal Online	373 306 67	(74.2) (82.0) (18.0)	370 285 85	(71.8) (77.0) (23.0)	RR^a^ 1.03 (0.96 to 1.11)
Secondary outcomes					
Questionnaire returned without chasing by study team Postal Online	259 207 52	(51.5) (79.9) (20.1)	217 161 56	(42.1) (74.2) (25.8)	RR^a^ 1.22 (1.07 to 1.39)
Total cost of vouchers, £	3790		1530		MD 4.56 (4.02 to 5.11)
Cost of vouchers per participant, £ – mean {SD}	7.53	{4.31}	2.97	{4.57}	

^a^Adjusted for centre

*SD* standard deviation, *RR* risk ratio, *MD* mean difference

Values are numbers (percentages) unless stated otherwise

**Fig. 2:**
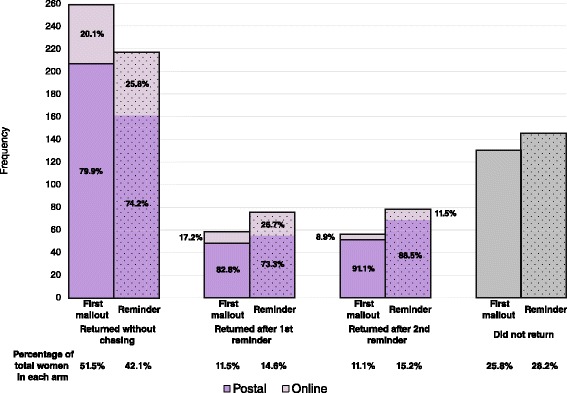
Return rates by number of reminder letters sent and method of completion

Figure 2 presents the percentage of questionnaires returned according to how many times a reminder letter was sent, and broken down by postal versus online completion. If a reminder was sent fewer women returned the questionnaire in the group receiving the promise of an incentive in the first mailout compared to those receiving a promise in the reminder letter (11.5 vs. 14.6 % for the first reminder, and 8.9 vs 11.5 % for the second reminder), with consistently more women completing the questionnaire online in the reminder group.

**Fig. 3:**
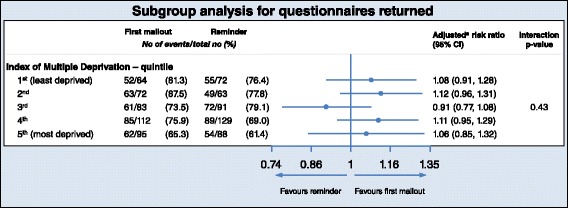
Subgroup analysis for questionnaires returned by IMD quintile

The pre-specified subgroup analysis is presented as a forest plot in Fig. 3. There is no evidence of heterogeneity for IMD subgroups for the primary outcome of overall response rate (*p* = 0.43), suggesting no differential intervention effect across deprivation quintiles.

## Discussion

In this study within a trial, there is no evidence to suggest that an offer of a monetary incentive at first mailout compared to only when a reminder letter is sent makes a substantial difference to the overall return rate of a 1-year follow-up questionnaire. Although there were slightly more questionnaires returned in the group receiving the offer at first mailout (an absolute difference of 3.4 %) the corresponding adjusted risk ratio of 1.03 (95 % CI 0.96 to 1.11) was not statistically significant at the 5 % level.

The return rate for women included in the SWAT compared to that before the SWAT was introduced demonstrated a marked improvement (absolute difference 17 %). Although this is not a randomised comparison, it is consistent with that found by Kenyon et al. [4] in a randomised study within a trial which showed an increase in the return rate of 11.7 % (95 % CI 4.7 % to 18.6 %) with the inclusion of a high-street voucher vs no voucher sent with a reminder letter to parents of seven year old children. In addition, a systematic review [2] including the study by Kenyon et al., showed that the addition of a monetary incentive was more effective than no incentive at increasing response rates to postal questionnaires (RR 1.18, 95 % CI 1.09 to 1.28).

This SWAT used a £10 high-street gift voucher as a monetary incentive. The mean cost of vouchers per participant was greater in the group receiving the offer at first mailout (£7.50 vs £3.00). Coupled with the lack of evidence of a difference in the overall return rate, this would indicate that sending the offer of an incentive with a reminder letter only is a cost-effective approach to improving return rates. However, there is evidence to suggest that the return rate without requiring reminders is higher in the group for whom the incentive is offered in the first mailout (absolute difference 9.4 %). The cost of administering the additional reminder letters was not calculated, but is a serious consideration that would need to be offset against the expected cost of the vouchers and could depend on the administration resources available in the trial team as well as the sample size of the study.

There are ethical issues to consider with the approach of only sending an offer of an incentive to those participants who do not return their questionnaire promptly. Consideration should be given to the chance that participants in a study may communicate with each other, and share their experiences regarding whether or not they received an incentive.

## Conclusion

This is the first known SWAT to investigate the use of incentives for improving questionnaire return rates in a population of first time mothers with infants around 1 year old. This study suggests that offering a monetary incentive when a reminder is required could be cost-effective depending on the sample size of the study and the resources available to administer the reminder letters.

## Abbreviations

CI, confidence interval; IMD, index of multiple deprivation; IQR, interquartile range; RR, risk ratio; SD, standard deviation; SWAT, study within a trial
